# Supplementary material for: Inflammatory breast cancer: a model for investigating cluster-based dissemination
Source: NPJ Breast Cancer. 2017 Jun 6;3:21. doi: 10.1038/s41523-017-0023-9 (PMC5460282; doi:10.1038/s41523-017-0023-9)
Supplement: Supplementary file 1 — Supplementary Information [file 41523_2017_23_MOESM1_ESM.docx]

**Inflammatory Breast Cancer: a model for investigating cluster-based dissemination**

Mohit Kumar Jolly, Marcelo Boareto, Bisrat G Debeb, Nicola Aceto, Mary C Farach-Carson, Wendy A Woodward, Herbert Levine

**Supplementary Information**

**Mathematical model construction**

This circuit contains the following components – miR-200 ($\mu_{200}$), ZEB mRNA ($m_{Z}$), ZEB protein ($Z$), miR-205 ($\mu_{205}$), and SNAIL (S). All of them have an innate production and degradation rate. The effects of miR-200 on ZEB are captured by both the degradation of mRNA by miRNAs (depicted by $Y_{m}(\mu)$) and the inhibition of translation by miRNAs (depicted by $L(\mu)$). Also, miRNAs that bind to mRNAs can be degraded after forming a complex with them (depicted by $Y_{\mu}(\mu)$). A detailed derivation of the functions is presented in our earlier work on devising a theoretical framework for microRNA-based circuits^1^.

*∆Np63α (P63)* is an external signal that activates miR-205 and SNAIL. ∆Np63α can increases the levels of mature miR-205 two-to-three fold^2,3^, hence it is assumed to have two binding sites on miR-205 that represses ZEB via binding to two sites in its 3’ UTR^4^. Further, ∆Np63α can activate SLUG (SNAI2)^2^ expression; this interaction is assumed to happen via one site. Transcriptional regulation is denoted by shifted Hill functions $H^{S}(X,\lambda)$defined as$H^{S}(X,\lambda)=H^{-}(X)+\lambda H^{+}(X)$, a weighted sum of positive and negative Hill functions. Parameter $\lambda$ in the shifted Hill functions is the weight factor that represents fold-change in production rate from its basal level, due to the binding of regulatory factor. For activation, $\lambda>1$ and shifted Hill function is represented by ; for repression,$0<\lambda<1$ and shifted Hill function is represented by; and for no change, $\lambda$=1.$\lambda_{X,Y}$denotes the effect of X on Y^1^.

The dynamics of miR-200 ($\mu_{200}$) can be described by the following equation:

where $g_{\mu_{200}}$ and $k_{\mu_{200}}$ are the innate production and degradation rates of miR-200 respectively. $H^{S-}\left(Z,\lambda_{Z,\mu_{200}}\right)$ represents the transcriptional inhibition of miR-200 by ZEB and $H^{S-}\left(S,\lambda_{S,\mu_{200}}\right)$ represents transcriptional inhibition of miR-200 by SNAIL. $Y_{\mu}\left(\mu_{200}\right)$ represents the degradation rate of miR-200 due to forming a complex with ZEB mRNAs.

The dynamics of ZEB mRNA ($m_{Z}$) and ZEB protein ($Z$) are described by the following equations:

$$\frac{dm_{Z}}{dt}=g_{m_{Z}}H^{S-}\left(\mu_{205},\lambda_{\mu_{205},m_{z}}\right)H^{S+}\left(Z,\lambda_{Z,m_{Z}}\right)H^{S+}\left(S,\lambda_{S,m_{Z}}\right)-m_{Z}Y_{m}\left(\mu_{200}\right)-k_{m_{Z}}m_{Z}.............(2)$$

$$\frac{dZ}{dt}=g_{Z}m_{Z}L\left(\mu_{200}\right)-k_{Z}Z\cdots\cdots\left(3\right)$$

where $g_{m_{Z}}$ and $g_{Z}$ are the innate production rates of ZEB mRNA and ZEB protein respectively, and $k_{m_{Z}}$ and $k_{Z}$ are their respective innate degradation rates. $H^{S+}\left(Z,\lambda_{Z,m_{Z}}\right)$ denotes transcriptional self-activation of ZEB, and $H^{S+}\left(S,\lambda_{S,m_{Z}}\right)$ denotes transcriptional activation of ZEB by SNAIL. $Y_{m}\left(\mu_{200}\right)$ represents the degradation of ZEB mRNA due to forming mRNA-miRNA complexes with miR-200, $L\left(\mu_{200}\right)$ denotes the translational inhibition of ZEB by miR-200. Members of the miR-200 family can have 6 or more binding sites on ZEB, and ZEB can repress miR-200 by 3 binding sites in its promoter region^1,5^.

The dynamics of miR-205 and SNAIL are given by equations (19) and (20) respectively:

$$\begin{matrix} \frac{d\mu_{205}}{dt}=g_{\mu_{205}}H^{S+}\left(P63,\lambda_{P63,\mu_{205}}\right)-k_{\mu_{205}}\mu_{205}\cdots\cdots\left(4\right) \\ \frac{dS}{dt}=g_{S}H^{S+}\left(P63,\lambda_{P63,S}\right)H^{S+}\left(X,\lambda_{X,S}\right)-k_{S}S\cdots\cdots\left(5\right) \end{matrix}$$

where $g_{\mu_{205}}$and $k_{\mu_{205}}$ denote the production and degradation rates for miR-205 respectively, and $g_{S}$ and $k_{S}$denote the production and degradation rates for SNAIL. $H^{S+}\left(P63,\lambda_{P63,\mu_{205}}\right)$and $H^{S+}\left(P63,\lambda_{P63,S}\right)$denote respective activation of miR-205 and SNAIL by *∆Np63α (P63) .*$H^{S+}\left(X,\lambda_{X,S}\right)$represents the activation of SNAIL by external signal X.

To derive the equations for the control case (i.e. Figure 3A) without *∆Np63α and miR205*, $\lambda_{\mu_{205},m_{z}}$and $\lambda_{P63,S}$ are set to 1.

The estimation of the levels for miRNAs, mRNAs, and proteins are made according to their canonical concentrations in eukaryotic cells. Typically, the volume of a mammalian cell is 100-10000 um^3^ and the concentration for a single protein is 10nM-1μM ^6^. 1μM protein concentration amounts to around 6 million number of molecules ($6.02\times10^{23}\times10^{-6}\times\left(10000\times\left(10^{-5}\right)^{3}\right)$ ). The ratio of protein/mRNA of one gene is about 2800^7^, hence the number of mRNA molecules for one gene should be around 1000. Number of microRNA molecules in a cell are around 10000 molecules^8^, hence $\mu_{0}$=10000 molecules. The translation rate for one gene is around 140 proteins per mRNA per hour^7^, so we used 100 proteins per ZEB mRNA as its translation rate. Innate degradation rates of miRNAs, mRNAs, and proteins were selected based on their half-lives from experimental data. Typically, the half-life of mammalian proteins is about 10 hours^9^; therefore we selected 0.1 hour^-1^ as the innate degradation rate for ZEB. The half-life of mRNA is a few hours ^10^, so we chose 0.5 hour^-1^ as the innate degradation rate for ZEB mRNA. The innate degradation rate of miR-200 and miR-205 was selected as 0.05 hour^-1^, as miRNAs are more stable than mRNAs ^11,12^. For transcriptional regulation, the weight factors for shifted Hill function vary from 5 to 10 for activation, and from 0.5 to 0.1 for repression. All parameters ae given in Tables S1-3 as given below.

| **Species** | **Production rates (molecules/Hour)** | | **Degradation rates (Hour^-1^)** | |
| --- | --- | --- | --- | --- |
| miR-200 | $g_{\mu_{200}}$ | 2100 | $k_{\mu_{200}}$ | 0.05 |
| ZEB mRNA | $g_{m_{Z}}$ | 11 | $k_{m_{Z}}$ | 0.5 |
| ZEB protein | $g_{Z}$ | 100 | $k_{Z}$ | 0.1 |
| miR-205 | $g_{\mu_{205}}$ | 2000 | $k_{\mu_{205}}$ | 0.05 |
| SNAIL | $g_{S}$ | 18000* | $k_{S}$ | 0.125 |

**Table S1. The production and degradation rates of different species in the circuits**

*****SNAIL production rate is calculated as Translation rate*steady state mRNA levels as given in^1^.

| **Description** | **Fold change** | **Value** | **# binding sites** | **Value** | **Threshold** | **Molecules** |
| --- | --- | --- | --- | --- | --- | --- |
| Self-activation of ZEB | $\lambda_{Z,m_{Z}}$ | 7.5 | $n_{Z,m_{Z}}$ | 2 | $Z^{0}_{m_{z}}$ | 25000 |
| Inhibition of miR-200 by ZEB | $\lambda_{Z,\mu_{200}}$ | 0.1 | $n_{Z,\mu_{200}}$ | 3 | $Z^{0}_{\mu_{200}}$ | 220000 |
| Activation of ZEB by SNAIL | $\lambda_{S,m_{Z}}$ | 10 | $n_{S,m_{Z}}$ | 2 | $S_{m_{z}}^{0}$ | 180000 |
| Inhibition of miR-200 by SNAIL | $\lambda_{S,\mu_{200}}$ | 0.1 | $n_{S,\mu_{200}}$ | 2 | $S_{\mu_{200}}^{0}$ | 180000 |
| Activation of SNAIL by ∆Np63α | $\lambda_{P63,S}$ | 2 | $n_{P63,S}$ | 1 | $P63_{S}^{0}$ | 8000 |
| Activation of miR-205 by ∆Np63α | $\lambda_{P63,\mu_{205}}$ | 4 | $n_{P63,\mu_{205}}$ | 2 | $P63_{\mu_{205}}^{0}$ | 5000 |
| Inhibition of ZEB by miR-205 | $\lambda_{\mu_{205},m_{Z}}$ | 0.5 | $n_{\mu_{205},m_{Z}}$ | 2 |  | 10000 |
| Activation of SNAIL by X | $\lambda_{X,S}$ | 3 | $n_{X,S}$ | 2 | $X_{S}^{0}$ | 1000 |

**Table S2. The parameters used in different Hill functions for different circuits**

| **n (# of miRNA binding sites)** | 0 | 1 | 2 | 3 | 4 | 5 | 6 |
| --- | --- | --- | --- | --- | --- | --- | --- |
| $l_{i}$ **(hour^-1^)** | 1 | 0.6 | 0.3 | 0.1 | 0.05 | 0.05 | 0.05 |
| $\gamma_{mi}$ **(hour^-1^)** | 0 | 0.04 | 0.2 | 1 | 1 | 1 | 1 |
| $\gamma_{\mu i}$**(hour^-1^)** | 0 | 0.005 | 0.05 | 0.5 | 0.5 | 0.5 | 0.5 |

**Table S3. The parameters used in Y(u), Y(m) and L functions.**

**References**

1. Lu, M., Jolly, M. K., Levine, H., Onuchic, J. N. & Ben-Jacob, E. MicroRNA-based regulation of epithelial-hybrid-mesenchymal fate determination. *Proc. Natl. Acad. Sci. U. S. A.* **110,** 18144–9 (2013).

2. Dang, T. T., Esparza, M. A., Maine, E. A., Westcott, J. M. & Pearson, G. W. ∆Np63α promotes breast cancer cell motility through the selective activation of components of the Epithelial-to-Mesenchymal Transition program. *Cancer Res.* **75,** 3925–3935 (2015).

3. Tran, M. N. *et al.* The p63 Protein Isoform ∆Np63α Inhibits Epithelial-Mesenchymal Transition in Human Bladder Cancer Cells: Role of miR-205. *J. Biol. Chem.* **288,** 3275–88 (2013).

4. Gregory, P. A. *et al.* The miR-200 family and miR-205 regulate epithelial to mesenchymal transition by targeting ZEB1 and SIP1. *Nat. Cell Biol.* **10,** 593–601 (2008).

5. Brabletz, S. & Brabletz, T. The ZEB/miR-200 feedback loop--a motor of cellular plasticity in development and cancer? *EMBO Rep.* **11,** 670–7 (2010).

6. Milo, R., Jorgensen, P., Moran, U., Weber, G. & Springer, M. BioNumbers--the database of key numbers in molecular and cell biology. *Nucleic Acids Res.* **38,** D750–D753 (2010).

7. Schwanhäusser, B. *et al.* Global quantification of mammalian gene expression control. *Nature* **473,** 337–342 (2011).

8. Lim, L. P. *et al.* The microRNAs of Caenorhabditis elegans. *Genes Dev.* **17,** 991–1008 (2003).

9. Eden, E. *et al.* Proteome Half-Life Dynamics in Living Human Cells. *Science* **331,** 764–768 (2011).

10. Yang, E. *et al.* Decay rates of human mRNAs: correlation with functional characteristics and sequence attributes. *Genome Res.* **13,** 1863–1872 (2003).

11. Gantier, M. P. *et al.* Analysis of microRNA turnover in mammalian cells following Dicer1 ablation. *Nucleic Acids Res.* **39,** 5692–5703 (2011).

12. Khanin, R. & Vinciotti, V. Computational Modeling of Post-Transcriptional Gene Regulation by MicroRNAs. *J Comput Biol* **15,** 305–316 (2008).
